# The emergence of genome architecture and zygotic genome activation

**DOI:** 10.1016/j.ceb.2020.02.002

**Published:** 2020-06

**Authors:** Antoine Vallot, Kikuë Tachibana

**Affiliations:** 1 Institute of Molecular Biotechnology of the Austrian Academy of Sciences, Vienna Biocenter (VBC), Dr. Bohr Gasse 3, 1030, Vienna, Austria; 2 Department of Totipotency, Max Planck Institute of Biochemistry, Am Klopferspitz 18, 82152, Martinsried, Germany

**Keywords:** Chromatin structure, Cohesin, Zygotic genome activation, ZGA, Reprogramming, Zygote, Hi-C, Totipotency, Pioneer transcription factor, TAD, CTCF

## Abstract

The fusion of two transcriptionally silent gametes, egg and sperm, generates a totipotent zygote that activates zygotic transcription to support further development. Although the molecular details of zygotic genome activation (ZGA) are not well understood in most species, an emerging concept is that one or more pioneer transcription factors trigger zygotic transcription. Concomitantly, extensive changes in 3D chromatin organization occur during development. In this review, we discuss recent advances in understanding when and how genome architecture emerges in early metazoan embryos, how the zygotic genome is activated, and how these events might be coordinated. We also highlight some of the unknowns that may be critical to address in the future.

## Introduction

At the earliest stage of development, the single-cell embryo inherits the genomes of two terminally differentiated germ cells, the sperm, and the oocyte. The newly formed zygote has the developmental potential to generate all cell types and a complete organism, which is the strictest definition of totipotency. It is thought that achieving a state of totipotency requires extensive epigenetic reprogramming (reviewed in a study by Ladstatter et al [1]) and activation of the zygotic transcriptional program. Around the time of zygotic genome activation (ZGA), higher order chromatin structure is reorganized in the early embryo. However, it remains unclear to what extent these two processes are mechanistically and functionally related. Here, we will focus on mouse (*Mus musculus*) embryonic development and draw connections to other species, such as fruit fly (*Drosophila melanogaster*) and zebrafish (*Danio rerio*). In these species, the proteins that trigger ZGA have been identified, whereas the drivers of 3D chromatin organization remain unclear, revealing that there are major gaps in our knowledge of genome architecture and ZGA in any one organism.

## Key features of genome architecture

The emergence of 3D chromatin organization during embryonic development has recently been investigated using chromosome conformation capture–based Hi-C methods (reviewed in a study by Dekker et al. [2]). Briefly, interphase chromatin is organized into loops, topologically associating domains (TADs) and compartments. Chromatin loops are proposed to be generated by a mechanism of loop extrusion throughout the genome [3, 4, 5, 6, ∗∗7]. Extruded loops can only be inferred from genome-wide contact probability plots owing to heterogeneity in size and location in every cell [8,9]. On the other hand, loops that encounter a boundary element such as such as CCCTC-binding factor (CTCF) might become transiently stabilized, increase in abundance in certain positions, and can be visualized as corner peaks in bulk Hi-C heat maps [10,11]. The most striking feature of Hi-C data is TADs, in which genomic loci within one TAD are more likely to be in physical proximity than to loci in another TAD. There is emerging evidence that TADs reflect a population average of loops [12]. Different models have been proposed to explain the formation of TADs [4,5,13]. The loop extrusion model provides a plausible explanation for how distant loci are brought into close proximity in 3D space (see the review by L. Mirny [this issue]) for an extended discussion). Cohesin has been proposed to function as a loop extrusion factor in the interphase [3,4]. Consistent with this, cohesin is required for loop and TAD establishment and maintenance [8,14, 15, 16, 17]. Crucially, recent single-molecule assays have demonstrated that cohesin can generate DNA loops *in vitro* [7,18], providing strong evidence for a cohesin-mediated mechanism of loop extrusion. In addition, chromatin is segregated into at least two types of compartments, in which A compartments correlate with transcriptionally active and B compartments correlate with transcriptionally repressed domains. Loop extrusion and compartmentalization appear to be antagonistic [14, 15, 16,19] but the precise mechanism leading to compartment segregation is poorly understood.

Given these different aspects of genome architecture, it is important to consider these separately when investigating the timing of their establishment and function during embryonic development. The idea that the function of TADs is to regulate gene expression by facilitating enhancer–promoter interactions may have to be revised and could be relevant to specific loci but not others [20, 21, 22]. Importantly, it is unclear to what extent TAD appearance reflects a biological function beyond implying increased border insulation, or whether the critical processes are loop formation or dynamics. Indeed, the precise function of loops in the interphase chromatin remains unclear. Therefore, it is fascinating to consider that the technologies are available to study these aspects of genome architecture, but we are still largely ignorant of the biological relevance of the observed structures.

## Timing of genome architecture emergence

Studies from different organisms have yielded surprisingly diverse timings of genome architecture establishment during embryonic development. To know whether 3D chromatin organization arises *de novo* during embryonic development or is inherited from gametes, it is necessary to know the chromatin folding states of the oocyte, egg, and sperm. Mammalian oocytes arrest after meiotic recombination in prophase I, which is a G2 phase–like interphase state. The chromatin of germinal vesicle (GV) stage oocytes is organized into loops, TADs, and compartments [12]. After the meiosis I division, the egg arrests with condensed chromosomes in metaphase II. The chromatin organization of metaphase II chromosomes resembles that of mitotic chromosomes, which lack TADs and compartments [23, 24, ∗25, ∗26]. Therefore, maternal chromatin inherited from the egg to the zygote establishes higher order chromatin structure anew, similarly to cells exiting mitosis into G1 phase [27,72]. In contrast, sperm chromatin is highly compacted by protamines. It was therefore an unexpected finding that the atypical chromatin composition does not appear to interfere grossly with TADs and compartments [28,29]. In contrast to mouse sperm, human sperm do not harbor any detectable TADs, which may be related to species-specific differences in CTCF abundance in gametes [30].

Recent studies have improved our understanding of 3D chromatin reorganization that occurs after fertilization. Single-nucleus Hi-C (snHi-C) of isolated maternal and paternal nuclei from zygotes revealed that chromatin is organized into loops and weak TADs in G1 phase zygotes [12]. These were not visualized on classical heat maps owing to the sparsity of data but calculated as an average loop based on contact frequencies at known positions of loops in other cell types. Although paternal chromatin segregates into A and B compartments, maternal compartment segregation is remarkably weak, also by 3D fluorescence in situ hybridization [12]. This unexpected observation was independently corroborated by bulk Hi-C of early mouse embryos [25,26]. One explanation is that the kinetics of compartment establishment is parent-of-origin–specific and possibly related to asymmetries in epigenetic modifications and the timing of transcriptional activation. Alternatively, sperm chromatin compartmentalization could be directly inherited into the paternal chromatin of the zygote. Maternal compartmentalization increases in the 2-cell embryo, coincident with ZGA [25,26].

Recently, two additional compartment-like domains have been investigated in mouse gametes and embryos. Lamina-associated domains (LADs) can be considered as a subtype of B compartments with a subcellular localization at the nuclear envelope [31,32]. LADs are assembled after fertilization in the early embryo [33]. Ectopic expression of Kdm5b prevents paternal LAD formation, suggesting that remodeling of the histone modification H3K4me3 is involved in LAD establishment [33]. Another new type of compartment, the polycomb associating domains (PADs), is detectable in the mouse oocyte, zygote and 2-cell stage embryo. Polycomb associating domains are marked by H3K27me3 and are generated in a manner that depends on the polycomb group protein EED in zygotes [34]. Therefore, distinct epigenetic chromatin states may give rise to different compartmental domains.

**Figure 1:**
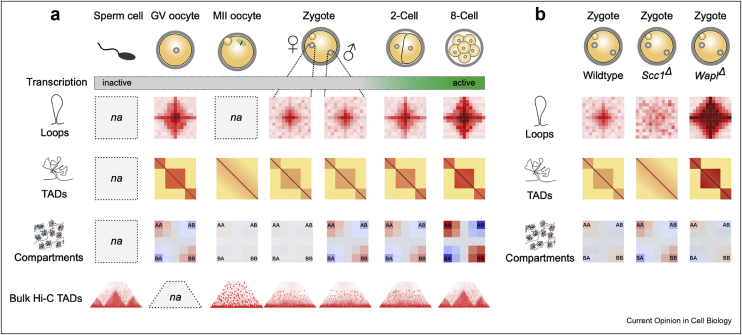
Emergence of genome architecture during early mouse development. (**a**) Schematic aggregate analysis of loops, TADs (topologically associating domains) and compartments during the oocyte-to-embryo transition [8,12]. Schematic heatmaps of TAD profiles from bulk Hi-C [25,26]. *na*: not available for single-cell Hi-C. Below each stage, transcriptional activity is displayed in green gradient. (**b**) Schematic aggregate analysis of combined loops, TADs and compartments in control, Scc1-cohesin conditional knockout and the cohesin-release factor Wapl (Wings apart–like protein homolog) conditional knockout zygotes [8].

Bulk and snHi-C studies of the first stages of embryonic development appear to yield conflicting results with regard to the timing of TAD formation. Bulk Hi-C heat maps of embryos do not reveal TAD profiles until embryos progress to the 8-cell morula stage (Figure 1). On the other hand, average loops and TADs are detected by snHi-C of zygotes. These contacts become undetectable in conditional genetic knockouts of cohesin and are enriched when cohesin release from chromosomes by Wapl is prevented [8], providing strong evidence that the average loops and TADs in zygotes reflect a biologically relevant mechanism, that is, loop extrusion. The differences in TAD timing can be explained by the sensitivity thresholds of different analyses. Reanalysis of bulk Hi-C data showed that average loops and TADs are also detectable in this data set in zygotes and become stronger during development [8]. Therefore cohesin-mediated loop extrusion commences from the earliest point after fertilization and loops can form weak TADs, before the major wave of ZGA at the 2-cell stage. TADs become progressively more pronounced during mouse embryonic development [8,25,26]. Importantly, recent work in human embryos has led to similar observations, with a gradual establishment of TADs and boundary consolidation that coincides with ZGA at the 4–8 cell stage [30].

The timing of TAD establishment is radically different in other species, in which it has not been experimentally addressed whether these domains are generated by cohesin-dependent loop extrusion. In *Drosophila*, CTCF does not grossly coincide with TAD borders, suggesting that these are generated by other proteins and/or mechanisms [35, 36, 37]. The rapidly dividing early embryo harbors a mostly unstructured genome until cycle 14, when TADs emerge and ZGA occurs [38]. In the Japanese rice fish Medaka (*Oryzias latipes*), a preprint reports that TADs and compartments are not detected before ZGA [39]. In contrast, domains are detectable from fertilization onward in zebrafish and, unexpectedly, appear to be transiently disassembled after ZGA [40]. Therefore, with the exception of zebrafish, TADs are either established at or become stronger features during ZGA.

## Mechanism of ZGA

The activation of zygotic gene expression is crucial for the development of an organism. Despite its importance, remarkably little is known about the mechanism of ZGA in most species, with some exceptions. In mammals, ZGA is characterized by a transient expression of transposable elements [41]. For instance, the murine endogenous retroviral element family expression peaks at the 2-cell stage in mouse embryos, concomitant with the major wave of ZGA [41]. Although their role during ZGA remains enigmatic, it has been recently proposed that transposable elements influence gene expression in mouse by altering chromatin accessibility [42].

**Figure 2:**
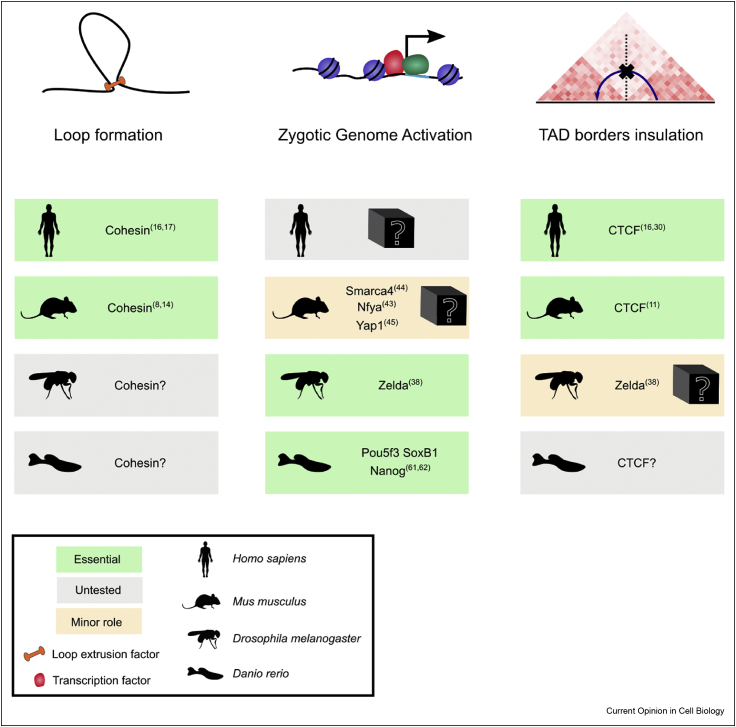
Selected summary of regulators of ZGA, loops and TAD border insulation in human (*Homo sapiens*), mouse (*Mus musculus*), fruit fly (*Drosophila melanogaster*) and zebrafish (*Danio rerio*) development. Essential proteins for each process are highlighted in green, proteins that are known to be present but have not been experimentally tested are highlighted in gray, and proteins with tested minor roles are highlighted in beige. ZGA, zygotic genome activation; TADs, topologically associating domains.

An emerging concept is that one or more pioneer transcription factors bind to closed chromatin, remodel its accessibility, and either directly initiate transcription or prime chromatin for other transcription factors to trigger transcription (Figure 2). In mouse, two of these transcriptional activators are likely NFYa and Smarca4. Both contribute to activation of a subset of genes at the major wave of ZGA and are essential for progression to the blastocyst stage [43,44]. The yes-associated protein 1 (Yap1) is also required to regulate a subset of genes during ZGA in mouse [45]. The Dux family of proteins have been proposed to function as pioneer factors in mouse and human as they are required to convert mouse embryonic stem cells to 2-cell embryo–like (2C-like) cells, which have some transcriptional similarities such as endogenous retroviral element activation to 2-cell stage embryos [46, 47, 48]. However, Dux knockout mice are viable and fertile, demonstrating that Dux is dispensable for ZGA in vivo [49,50]. Studies in 2C-like cells have shown that Dux is regulated by Dppa2/4 [51, 52, 53], and it will be interesting to see whether these regulators are essential for ZGA and development *in vivo*. Therefore, the master regulators of genome activation are largely unknown in mammals.

The pioneer transcription factor Zelda is essential for ZGA in *Drosophila* [54]*.* Zelda is maternally deposited as mRNA in the egg and translated into a six CH2H2 zinc finger-binding domain protein after fertilization [54,55]. Zelda binding to its motif is required for chromatin opening and ZGA [55, 56, 57]. In accordance with the biochemical definition [58], Zelda has pioneer factor activity because it can bind nucleosomes *in vitro* [59]. Interestingly, Zelda appears not to be conserved outside insects [60]. Therefore, ZGA in other species is triggered either by unrelated pioneer factors or by distinct mechanisms.

Additional insights into how ZGA might be orchestrated in vertebrates come from zebrafish studies. The combined loss of the transcription factors Nanog, Sox19b, and Pou5f3 (Oct4) prevents transcription of >75% of genes activated at ZGA and causes gastrulation failure [61,62]. All three proteins are required for maintaining pluripotency in mouse embryonic stem cells [63], but whether they are required for ZGA and have pioneer factor activity are not known. They might be unlikely candidates for mammalian ZGA as Nanog is expressed later during development and conditional genetic knockout or knock-down of Oct4 does not prevent ZGA [64,65]. However, a similar triple manipulation experiment has not been reported for mouse. Therefore, multiple transcription factors might cooperate to trigger ZGA in mammals.

## Coordination of 3D chromatin organization and ZGA

The key question is how genome architecture and ZGA are mechanistically related. Is chromatin organization a prerequisite for ZGA? In zebrafish, cohesin depletion in early embryos leads to a delay in ZGA many cell cycles later [66], which could be due to either direct or indirect effects. It is also not known whether chromatin organization is perturbed under these conditions. In mouse, cohesin depletion leads to a zygotic arrest due paternal chromatin reprogramming and checkpoint activation, precluding a straightforward analysis of ZGA [67]. Experiments using acute protein degradation approaches that prevent loop/TAD formation, which have been informative for studying higher order chromatin structure in cell culture [14, 15, 16], might shed further light into whether these structures are required for efficient ZGA.

A related question is whether transcription is required for higher order chromatin structures. With the exception of human embryos, transcription is not required for loop and TAD formation in embryos [12,25,26,30,38]. Specifically, α-amanitin treatment inhibits ZGA but does not prevent TADs in mouse and fruit fly embryos [25,26,38]. It is conceivable that RNA polymerase II recruitment results in collisions with the loop extrusion machinery and possibly generates boundaries [68]. Because α-amanitin causes degradation of the RPB1 subunit of RNAPII with drug dosage–dependent kinetics [69], the extent of residual chromatin-bound RNAPII might lead to vestiges of domains.

However, loops are detectable in transcriptionally silent mouse zygotes and TADs are detectable pre-ZGA in zebrafish [8,12,40], suggesting that transcription is dispensable for these structures during unperturbed development. However, a striking exception appears to be the human embryo, where ZGA is required for TAD formation [30]. It is interesting to consider what might underlie this difference between human and other organisms. In mouse, CTCF is detected in oocytes and early embryos [65] and CTCF-anchored loops are detected in zygotes using snHi-C [12]. However, CTCF is undetectable in human embryos before ZGA, suggesting that embryonic CTCF expression is likely necessary for TAD border insulation. This does not directly mean that transcription is required for loops and TADs but rather that the machinery needed for the formation has to be produced by ZGA. Therefore, with the possible exception of human embryos, loops and TADs are generated independently of zygotic transcription, which is consistent with work in tissue culture cells [17].

Finally, the most striking change in chromatin organization associated with ZGA is an increase in TAD boundary insulation. Interestingly, the pioneer transcription factor Zelda (Figure 2) is required for locus-specific TAD boundary insulation [38], raising the possibility that transcription factor binding might be a conserved mechanism for boundary insulation in organisms lacking CTCF at boundaries. In mouse, the mechanism leading to increased TAD boundary insulation at ZGA is unclear. We hypothesize that an increase in CTCF occupancy and/or longer cohesin residence time will result in pausing of the cohesin complex at boundaries. What still remains an open question is whether a change in TAD boundary insulation has a biological function or is a by-product of the loop extrusion machinery encountering a more complex chromatin environment.

## Future perspectives

Rapid progress has been made in the last two years in describing the emergence of genome architecture during development by genomic methods in several organisms. By comparison, ZGA was first described decades years ago in vertebrates [70,71], but our understanding of this fundamental process is still limited to very few organisms. By overlapping these different knowledge areas, it becomes apparent that we do not have a complete understanding of either in any one organism. In mammals, we are largely ignorant of the essential transcription factors that trigger ZGA. In most nonmammalian species, functional evidence for proteins required for TAD formation and boundary insulation is needed. Filling in these unknowns over the next years will illuminate to what extent the principles of ZGA and related changes in genome architecture are conserved throughout metazoan evolution.

## Credit author statement

Both authors A.V. and K.T. wrote and edited the manuscript.

## Conflicts of interest statement

Nothing declared.
